# Therapeutic anticoagulation can be safely accomplished in selected patients with traumatic intracranial hemorrhage

**DOI:** 10.1186/1749-7922-7-25

**Published:** 2012-07-23

**Authors:** Matthew C Byrnes, Eric Irwin, Robert Roach, Molly James, Patrick K Horst, Patty Reicks

**Affiliations:** 1 Department of Trauma, North Memorial Medical Center, Robbinsdale, MN, USA; 2 Division of Critical Care and Acute Care Surgery, University of Minnesota, Minneapolis, MN, USA; 3 North Memorial Medical Center, Division of Trauma, 3300 Oakdale Avenue, Robbinsdale, MN 55422, USA

## Abstract

**Introduction:**

Therapeutic anticoagulation is an important treatment of thromboembolic complications, such as DVT, PE, and blunt cerebrovascular injury. Traumatic intracranial hemorrhage has traditionally been considered to be a contraindication to anticoagulation.

**Hypothesis:**

Therapeutic anticoagulation can be safely accomplished in select patients with traumatic intracranial hemorrhage.

**Methods:**

Patients who developed thromboembolic complications of DVT, PE, or blunt cerebrovascular injury were stratified according to mode of treatment. Patients who underwent therapeutic anticoagulation with a heparin infusion or enoxaparin (1 mg/kg BID) were evaluated for neurologic deterioration or hemorrhage extension by CT scan.

**Results:**

There were 42 patients with a traumatic intracranial hemorrhage that subsequently developed a thrombotic complication. Thirty-five patients developed a DVT or PE. Blunt cerebrovascular injury was diagnosed in four patients. 26 patients received therapeutic anticoagulation, which was initiated an average of 13 days after injury. 96% of patients had no extension of the hemorrhage after anticoagulation was started. The degree of hemorrhagic extension in the remaining patient was minimal and was not felt to affect the clinical course.

**Conclusion:**

Therapeutic anticoagulation can be accomplished in select patients with intracranial hemorrhage, although close monitoring with serial CT scans is necessary to demonstrate stability of the hemorrhagic focus.

## Introduction

Injury represents one of the most common causes of morbidity and mortality in children and young adults. Although many complications can be seen after injury, venous thromboembolic disease can be among the most vexing. Virchow’s triad involves venous stasis, endothelial injury, and hypercoaguability, which are often seen in this patient population [1-3]. Injured patients often require immobility as a result of critical illness or skeletal fractures. Endothelial injuries are caused by fractures or venous stretching, and hematologic alterations associated with trauma result in hypercoagulability. The risk of venous thromboembolism (VTE) is dependent upon the specific injuries present in individual patients. While a single site arm fracture is unlikely to lead to VTE, a multisystem injury that includes a spinal cord injury, head injury, and multiple long bone fractures is very likely to lead to VTE [1]. The actual risks of VTE have been estimated to vary between 7%–58% [4].

A significant amount of study has been directed at preventing VTE in injured patients. Prophylactic doses of heparin or low molecular weight heparin have been demonstrated to significantly reduce the risk of VTE [4,5]. This intervention has been demonstrated to be safe within days of the initial injury, with only a small risk of bleeding complications. Once a thrombosis or embolus has occurred, however, prophylactic doses of anticoagulation are no longer adequate.

Injured patients are also at risk of arterial thromboembolism (ATE). Patients with mitral valve replacements are at risk of cerebrovascular accidents without anticoagulation. Patients with traumatic blunt cerebrovascular injury are also at risk without anticoagulation.

The traditional treatment of VTE has been therapeutic levels of anticoagulation [3]. The primary complication of therapeutic anticoagulation is hemorrhage, which is a significant consideration in injured patients. Patients with intracranial hemorrhagic diatheses (traumatic and nontraumatic) have been felt to be at an especially high risk of developing complications of anticoagulation [2,6]. Extension of an intracranial bleed can be especially troublesome and can potential lead to death or severe disability. In the presence of a contraindication to anticoagulation, inferior vena cava filters have been recommended to prevent embolus of thrombi from the lower extremity venous system to the pulmonary vasculature [3]. While this approach is reasonable for many injured patients, there are certain patient populations who would benefit from anticoagulation. As such, it is important to know the risks of therapeutic anticoagulation in patients with intracranial hemorrhage. Unfortunately, there is very literature to guide clinical decisions. Expert recommendations have suggested that therapeutic anticoagulation should be avoided, but no studies to date have reported the safety profile of this intervention.

Herein, we developed a study with the following objectives: (1) to evaluate the likelihood of extension of intracranial bleeding after the introduction of therapeutic anticoagulation; and (2) to evaluate the time course associated with introduction of therapeutic anticoagulation after the initial injury.

## Methods

Medical records of patients admitted to a university affiliated Level I trauma center were reviewed. Patients who had both a thrombotic complication and an intracranial hemorrhage were selected for inclusion. The thrombotic events that were incorporated in the study included: deep venous thrombosis (DVT), pulmonary embolus (PE), and blunt cerebrovascular injury. Patient demographics and CT scan results were noted. Patients were stratified according to the decision to use therapeutic anticoagulation vs. another treatment modality. Mortality and expansion of hemorrhage on CT scan were compared between the groups.

All patients were admitted to the trauma service. All patients received a head CT on admission and neurosurgery was subsequently consulted. There were four trauma surgeons during the study period that served as the core of the program and there were two neurosurgeons that were consulted on all patients with neurologic injuries. Patients who had leg swelling or unexplained hypoxia were evaluated for DVT or PE. This was done with bedside sonography and CT angiography. During the study period, we did not perform screening sonography, so all the DVT in the study were initially suspected based upon symptoms. We currently screen patients who do not receive prophylactic anticoagulation every four days, but this protocol was developed after this study was completed. We developed a formal screening criterion to evaluate for blunt cerebrovascular injury during the study time period. These criteria included a fracture of C1 through C4, LeFort 3 fracture, unexplained neurologic deficit, and fracture through the vascular foramen.

All patients in this study were regularly discussed with the neurosurgical service. When a diagnosis of DVT, PE, or blunt cerebrovascular injury was made, a discussion was held regarding the appropriateness of anticoagulation. After reviewing the radiologic images and the clinical course, the neurosurgeon determined whether or not anticoagulation could be safely administered. These decisions were made on a case by case basis. There was not a specific protocol for obtained follow up head CT scans after anticoagulation was started, but this was typically done 1–4 days later.

Data were analyzed with Analyse-It (Leeds, England). Categorical data were analyzed with chi-square tests and continuous data were analyzed with t-tests. Permission to conduct the study was obtained from the institutional review board at North Memorial Medical Center, which includes an ethical review of the research protocol.

## Results

During the study period, there were 42 patients who had both an ICH and an indication for anticoagulation. The average patient age was 50 years. 31% were female. The average injury severity score was 30.7.

Patients who received therapeutic anticoagulation were compared with patients who were treated without anticoagulation (Table 1). Twenty-six patients received anticoagulation, and 16 patients were treated without anticoagulation. The average age was similar in both groups. The gender distribution was identical in each group. The average length of stay was higher in the patients receiving anticoagulation (30 days vs. 20.9 days, p = 0.01). The thrombotic events were primarily composed of DVT and PE, with two cases of blunt cerebrovascular injury in each group.

**Table 1:** Patient characteristics

	**Anticoagulation**	**No Anticoagulation**	**p**
N	26	16	
Mean Age	51	48	0.43
Gender**			
--M	18 (69%)	11 (69%)	1.0
--F	8 (31%)	5 (31%)	
Mean ISS	31.1	30.1	0.95
Mortality	2 (7.7%)	2 (12.5%)	0.63
Mean LOS	30.0	20.9	0.01
Thrombosis*			
--PE	16	8	0.53
--DVT	15	9	1.0
--BCVI	2	2	0.63

*some pts had more than one type of thrombosis (DVT and PE). Blunt cerebrovascular injury (BCVI).

As noted by the high injury severity scores, most of the patients had significant injuries beyond the traumatic head injury. Concomitant injuries included 16 patients with skull fractures, 17 with spinal cord injuries, 8 with long bone fractures, 20 with at least one known rib fracture, 2 blunt liver injuries and 5 splenic injuries.

Overall, 62% of patients received therapeutic anticoagulation for treatment of their thrombotic complication (Table 2). All patients receiving anticoagulation received either enoxaparin at a dose of 1 mg/kg BID or a heparin drip with a goal PTT between 60 and 80 s (our high intensity protocol). The average time to instituting anticoagulation was 11.9 days after admission. Nearly one-quarter of the patients received full anticoagulation within the first 7 days of admission. Among these patients, two were anticoagulated within 24 h of injury, two were anticoagulated on day 4, and two were anticoagulated on day 6. Approximately 30% of patients were not anticoagulated until two weeks after their injury.

**Table 2:** Anticoagulation characteristics


Percent receiving anticoagulation	62%
Mean time until anticoagulation	11.9 days (range: 0–24)
Percent <7 days	23.1%
Percent 7–14 days	46.2%
Percent >14 days	30.7%

The decision to anticoagulate was not protocolized. Rather, the decision was left to the discretion of the attending neurosurgeon, in discussion with the trauma surgeon. The distribution of intracranial hemorrhage is listed in Table 3. The frequency of epidural, subdural, and intraparenchymal hemorrhage was similar between the groups. The average size of extra-axial hemorrhage was 9.48 mm in the group receiving anticoagulation and 9.89 mm in the group that did not receive anticoagulation. There was not a difference in rate of craniotomy for the treatment of the intracranial hemorrhage between the groups (30.8% vs. 56.6%, p = 0.19).

**Table 3:** Decision to anticoagulate

	**Anticoagulation**	**No Anticoagulation**	**p**
Epidural	1	2	0.54
Subdural	13	9	0.75
SAH	20	13	1.0
Contusion	14	12	0.21
Marshall Score			

There was extension of intracranial hemorrhage after institution of anticoagulation in only one patients. 96% of patients had no change in the volume of intracranial bleeding after initiation of anticoagulation. The extension of bleeding was very minor in one patient (1-2 mm growth in intraparenchymal hemorrhage), and the clinical course was felt to be unaffected. This was noted on follow up imaging 6 days after initiation of anticoagulation.

There were two deaths in each group of patients. The causes of death related to brain injury and multisystem organ failure. There were no deaths strictly from the thrombotic complications.

## Discussion

Injured patients are at significant risk of both hemorrhagic and thrombotic complications. These divergent risks create a therapeutic conundrum for trauma surgeons. Use of anticoagulation can lead to potential exsanguination and death, while avoidance of anticoagulation can lead to thrombotic complications and death [7]. Our data represents a novel report that suggests that therapeutic anticoagulation can be safely accomplished in select patients with intracranial hemorrhage.

There is very little to guide trauma surgeons in the safety profile of therapeutic anticoagulation. A recent review by Golob, et. al. evaluated the safety of initiating therapeutic anticoagulation in multi-injured trauma patients [7]. They noted that 21% of patients had complications from the therapy. The most common complication was an acute drop in hemoglobin requiring a blood transfusion; three patients died as a result of hemorrhage. Clinical factors associated with a higher risk of complications were COPD, low platelet count before therapy, and the use of unfractionated hemorrhage. This study, however, did not include any patients with head injuries, so extrapolation to this population is difficult.

Injured patients are at significant risk of thrombotic complications. Patients with multisystem trauma may develop DVT at a rate of 58%, while a quarter of patients with isolated intracranial hemorrhage may develop DVT [1]. This has led to significant study evaluating medical DVT prophylaxis in head injured patients. These studies have evaluated both low dose heparin and low molecular weight heparin. Norwood, et.al. noted that enoxaparin could be safely administered to select patients within 24 h of craniotomy for trauma [8]. In a separate report, this group noted a 3.4% progression rate of intracranial hemorrhage after institution of prophylactic doses of anticoagulants [2].

These reports were highly important in that they dispelled the traditional viewpoint that prophylactic anticoagulation is unsafe after brain trauma. They do not, however, speak to the safety profile of therapeutic anticoagulation. Traditional recommendations suggest that therapeutic anticoagulation is unsafe after traumatic intracranial hemorrhage. Textbooks have noted that anticoagulation should be delayed for 3 days to 6 weeks after injury “depending on local customs” (although no references were cited to support this recommendation) [9]. Our data suggests that anticoagulation in the earlier portion of this window may be safe.

Much of the hesitation to use therapeutic anticoagulation after brain trauma likely stems from studies on pre-injury use of anticoagulants. Cohen, et.al. reported mortality rates of 84%–91% among patients who were anticoagulated prior to an intracranial bleed [10]. Mina, et.al. compared anticoagulated patients to matched controls and found an absolute increase in mortality of 30% among the anticoagulated patients [11]. Another study evaluated the effect of rapid reversal of coagulopathy. Patients who underwent a rapid, protocolized reversal of coagulopathy had a 38% absolute reduction in mortality compared to historical controls [12]. Although these studies clearly indicated higher risks of death and disability among patients exposed to anticoagulants before the time of injury, they do not speak to the risks of administration of anticoagulants in a delayed fashion.

While many thrombotic complications can be treated without anticoagulation, there are specific scenarios in which anticoagulation has the potential to markedly improve a treatment regimen. Inferior vena cava (IVC) filters are the mainstay of treatment of both DVT and PE in patients with a contraindication to anticoagulation [3]. There are certain situations, however, in which IVC filters are not adequate. The filters do not prevent propagation of a thrombus that has already embolized to the pulmonary vasculature. A saddle PE requires very little propagation to result in lethal shock, so anticoagulation in this population is critical. Similarly, the long term morbidity of phlegmasia cerulean dolens is reduced with anticoagulation. Further, there is a small, but defined, risk of thrombosis of the IVC after placement of a filter [6]. This situation also requires anticoagulation. A final venous thrombosis that that is not amenable to treatment with an intravascular filter is an upper extremity DVT. Superior vena cava filters are uncommon and would lead to fatal intracranial swelling in the event of filter thrombosis.

There is only one report that has attempted to define the optimal treatment regimen of DVT or PE after intracranial hemorrhage [6]. This report focused on non-traumatic hemorrhage, so the generalizability may be limited. The authors conducted a review of the literature and were unable to develop firm recommendations.

Blunt cerebrovascular injury is another event that may require anticoagulation despite the presence of an intracranial hemorrhage [13]. Dissection of the carotid or vertebral arteries can lead to disabling or fatal stroke events, which may be prevented by adequate anticoagulation. Although much of the focus of treatment has shifted to antiplatelet regimens, there is a role for heparin in select cases. Our data suggests that therapeutic anticoagulation can be safely given to select patients with blunt cerebrovascular injury and intracranial hemorrhage.

Patients with mechanical cardiac valves represent a significant challenge to trauma surgeons [14-17]. The risk of artificial valves appears to be the highest in patients with a cage/ball valve in the mitral position. Atrial fibrillation and reduced left ventricular function add to the risk of stroke without anticoagulation. The natural history of these patients is unclear, as they are generally on anticoagulants, but we can glean some estimate of risk from studies that have evaluated temporarily discontinuing anticoagulation after intracranial hemorrhage. It appears safe to discontinue anticoagulation for brief periods of time [14,15]. Most of this work has been conducted in patients with spontaneous intracranial hemorrhage. It is possible that traumatic hemorrhage is a different entity, as injured patients are more hypercoaguable than then general population. Our data represents an important adjunct to these studies, in that we have demonstrated that early reintroduction of anticoagulation can be safely accomplished.

There are limitations of this study worth noting. We did not have a protocolized approach to management of anticoagulation. Rather, we consulted with the neurosurgeons on a daily basis and we started anticoagulation when their clinical judgment indicated it was safe to do so. As such, we are likely dealing with a highly select patient population. Additionally, our sample size is limited. It is possible that we would have yielded different results with a larger sample size. Finally, some of our patients received anticoagulation for uncomplicated PE rather than the extreme examples listed in this discussion. This does not detract from our results demonstrating safety of anticoagulation, however.

In conclusion, selected patients with brain injury may safely be anticoagulated to prevent the propagation of thrombotic complications. Our data does not provide definitive evidence of the safety profile. Rather, this manuscript provides initial evidence that suggests that traditional beliefs about anticoagulation in patients with brain injuries may be incorrect. Our data should be used a springboard to develop further study on this issue, so that the specific groups that would most benefit from anticoagulation could be defined.

## Competing interests

None of the authors have any conflicts of interest or special declarations to make regarding the contents of this manuscript.

## Authors’ contribution

MB directed the design of the study, data interpretation, and was involved in the drafting and revision of the manuscript. EI was involved in the study design and the manuscript revision. PR was involved in the data acquisition, study planning, and manuscript revision. RR was involved in the data interpretation and manuscript revision. PH was involved with the data acquisition and the data interpretation. All authors read and approved the final manuscript.
